# Common Mental Disorders Associated with Tuberculosis: A Matched Case-Control Study

**DOI:** 10.1371/journal.pone.0099551

**Published:** 2014-06-17

**Authors:** Gleide Santos de Araújo, Susan Martins Pereira, Darci Neves dos Santos, Jamocyr Moura Marinho, Laura Cunha Rodrigues, Mauricio Lima Barreto

**Affiliations:** Departamento de Saúde Coletiva, Institute of Collective Health of the Federal University of Bahia, Bahia, Brazil; 2 Departamento de Medicina Interna, School of Medicine and Public Health, Bahia, Brazil; 3 Department of Epidemiology and Population Health, London School of Hygiene and Tropical Medicine, London, United Kingdom; Fundació Institut d’Investigació en Ciències de la Salut Germans Trias i Pujol. Universitat Autònoma de Barcelona. CIBERES, Spain

## Abstract

**Introduction:**

Despite the availability of treatment and a vaccine, tuberculosis continues to be a public health problem worldwide. Mental disorders might contribute to the burden of the disease.

**Objective:**

The objective of this study was to investigate the association between common mental disorders and tuberculosis.

**Methods:**

A matched case-control study was conducted. The study population included symptomatic respiratory patients who attended three referral hospitals and six community clinics in the city of Salvador, Brazil. A doctor’s diagnosis defined potential cases and controls. Cases were newly diagnosed tuberculosis cases, and controls were symptomatic respiratory patients for whom tuberculosis was excluded as a diagnosis by the attending physician. Cases and controls were ascertained in the same clinic. Data collection occurred between August 2008 and April 2010. The study instruments included a structured interview, a self-reporting questionnaire for the identification of common mental disorders, and a questionnaire for alcoholism. An univariate analysis included descriptive procedures (with chi-square statistics), and a multivariate analysis used conditional logistic regression.

**Results:**

The mean age of the cases was 38 years, and 61% of the cases were males. After adjusting for potential confounders, the odds of tuberculosis were significantly higher in patients dignosed with a common mental disorder (OR: 1.34; 95% CI 1.05–1.70).

**Conclusion:**

There appears to be a positive and independent association between common mental disorders and tuberculosis; further epidemiological studies are required to increase our understanding of the possible biological and social mechanisms responsible for this association. Independent of the direction of the association, this finding has implications for the provision of care for mental disorders and for tuberculosis.

## Introduction

There are 8.6 million new cases of tuberculosis (TB) and 1.3 million deaths every year worldwide [1]. In recent years, there has been growing interest in co-morbidity between mental health and infectious diseases [2]. The global incidence of TB has declined slower than expected [3]. Mental disorders make a substantial independent contribution to the burden of disease worldwide [4]. Common mental disorders (CMDs) are characterized by a broad range of depressive, anxiety or somatoform symptoms, including irritability, insomnia, nervousness, fatigue and feelings of uselessness [5]. In developing countries, the prevalence of CMDs varies between 20%–30% [6]. CMDs have a chronic and disabling nature, cause intense subjective suffering and affect individuals’ abilities to care for their own health [5], [7].

An association has been described between TB and CMDs, where approximately 39%–70% of pulmonary TB cases have been found to have anxiety or depression [8]–[10]. A link between CMDs and tuberculosis is plausible, and if such a link exists, there are implications for the control and treatment of both diseases [2]. CMDs and TB are both associated with greater social vulnerability, inadequate living conditions and socioeconomic inequality [6], [11]. For patients with CMDs, the work capacity and self-image, and presumably the ability to adhere to an extended course of treatment, can be severely impaired [5], [7].

Multiple factors promote the onset of infectious diseases. These factors lead to increased exposure or accelerated progression from exposure to clinical diseases. Neuropsychiatric disorders are known to result in immune and hormonal dysfunctions, which may contribute to infectious diseases [12], [13]. Individuals with CMDs are frequent users of health services; these individuals often seek physical and psychological support [14]. Health services are often crowded and poorly ventilated environments, which increases the risk of exposure to infections [15]. Our study aimed to investigate whether there is an association between CMDs and pulmonary TB.

## Methods

### Study Area, Population and Design

This research was conducted in the city of Salvador (state of Bahia, Northeastern Brazil), which has an estimated population of 15 million [16]. TB treatment is available at no cost in the network of services of the Unified Health System. The main health centers that investigate respiratory symptoms in the city were included in this study, including the outpatient clinics of three referral hospitals and six primary health units. We conducted a case-control study with incident TB cases, paired by age (with 5 years of variation) and sex (1∶1 proportion). Treating tuberculosis as the outcome, for a significance level of 5%, 95% power, odds ratio of 1.5 and prevalence of CMDs (treated as the exposure) of 30%, the sample size was estimated to be 700 cases and 700 controls in an urban area of a city in Bahia, Brazil [17].

### Definition, Eligibility and Inclusion Criteria for Cases and Controls

Cases and controls were individuals living in Salvador who were older than 14 years and who presented with respiratory symptoms and were investigated for tuberculosis at any of several health care units. The individuals had no previous history of tuberculosis, and all individuals agreed to participate. Cases were individuals who were diagnosed with pulmonary TB by the attending chest physician. All potential cases underwent a smear microscopy and culture for *M. tuberculosis*, and individuals without a positive result and who did not live in Salvador were excluded from the cases. Controls were selected from the symptomatic respiratory patients who were excluded from a diagnosis of tuberculosis by the attending chest physician and who did not report active pulmonary TB in the past. All controls underwent a sputum and culture examination, and patients with a positive result and who did not live in Salvador were excluded from the controls. The first eligible control who was an acceptable age and sex match was recruited into the study.

### Data Collection Instruments

Data were collected by a team of nursing technicians under the supervision of senior nurses between August 2008 and April 2010. After the interview, samples of sputum were obtained from expectoration. The material was collected in sterile containers, properly stored in refrigerated boxes, and sent to the Central Laboratory of Public Health for smear microscopy and culture. This material was used for a molecular biology study that is not reported here.

A standardized questionnaire was used to collect clinical and socio-demographic data. The Self-Reporting Questionnaire (SRQ-20), a standardized instrument comprised of 20 questions, was used to identify CMDs. The SRQ-20 is a well-recognized instrument that is used to establish the presence or absence of a CMD. The instrument was validated in Brazil and achieved a sensitivity of 83% and a specificity of 80% [18]. The Cut Down, Annoyed, Guilty and Eye Opener (CAGE) questionnaire, also validated in Brazil, was used to identify alcohol abuse [19]. The instruments were applied before the doctor diagnosed the presence or absence of TB and, therefore, before treatment was initiated.

### Study Variables and Analysis

Data were collected using SPSS version 16 and analyzed using STATA version 11. We treated TB as the outcome (dependent variable) and a CMD as the independent variable, both categorized as present or absent. The SRQ-20 includes 20 symptoms that are aggregated in four categories (depressive/anxious mood, somatic symptoms, reduction in vital energy and depressive thoughts). We also investigated the association among the categories, the individual symptoms, and tuberculosis. Finally, the individual items on the SRQ-20 scale were summed, and individuals who scored above a cut-off of seven were considered to have a CMD [18]. The following co-variables were included: marital status (married, stable relationship/single, widower, divorced), ethnicity (white/non-white), history of contact with TB (yes/no), diabetes (yes/no), monthly family income (< the minimum salary (US $145)/≥ the minimum salary), type of residence (owned/not owned), number of household goods (radio, television, cooker, digital video disc player, refrigerator, landline, cellphone, washing machine, micro- wave oven, video camera; computer; car) (0–6 goods/≥7 goods), level of education (0–7 years/≥8 years), crowding (>1 person per room/≤1 person per room), recreational drugs (yes/no), smoking (yes/no), and alcohol consumption (dependent/not dependent). Respondents with two positive responses out of the four questions in the CAGE questionnaire were considered to be alcohol dependent.

The Person’s Chi-square test was used to evaluate the association between CMDs and TB. The association was estimated with odds ratios (OR) and their respective 95% confidence intervals using a conditional logistic regression model with backward stepwise procedures, conditional analysis for individual matching, unconditional for frequency matching. Variables that were associated with the outcome in the univariable analysis with p≤0.25 and those that contributed to the model’s goodness-of-fit, the matching variables (age and sex) and any apriori confounders were included in the model. The funders of the study did not require that the data be made available to other researchers; however, the principal investigators support data sharing and are happy to consider requests for data access.

### Ethical Considerations

The study was approved by the Ethics Committee of the Institute of Collective Health at the Federal University of Bahia, Brazil in Salvador (number 012-07). The committee determined that the study met ethical legal and regulatory norms and standards for research involving human subjects in Brazil as well as international norms and standards. Written informed consent was obtained from all patients of the study by a team of nursing technicians under the supervision of senior nurses; for adolescents between ages 14 and 18, written consent was signed by their parents or guardians. The security of the documents that were obtained during the study is the responsibility of the scientific team involved in the project. Patient information will be kept confidential, with exclusive access to the relevant project team members.

## Results

A total of 1,434 individuals participated in the study, of which 717 were TB cases and 717 were controls. There were no differences in the matching variables between the cases and controls (p>0.05). The majority of the cases were males (61.0%), with a mean age of 38.2 years (SD = 14.2 years) and a range of 15 to 92 years (Table 1).

**Table 1:** Distribution of pairing variables among tuberculosis cases and controls.

Variables	Cases (n = 717)			Controls (n = 717)			P-value
Sex**	N	%	Mean±SD*	N	%	Mean±SD*	
Male	437	61.0	-	434	60.5	-	0.87
Female	280	39.0	-	283	39.5	-	
Age group**			-			-	
14–29	241	33.6	-	238	33.2	-	0.99
30–44	234	32.6	-	238	33.2	-	
45–92	242	33.8	-	241	33.6	-	
**Age (in years)**			38.2±14.2			38.3±14.6	

City of Salvador, Brazil, 2008–2010.

*Standard deviation; **p>0.05.

Table 2 shows a predominance of “somatic symptoms” among the cases (40.9%) and control (41.6%). Nine of the 20 SRQ-20 symptoms were individually associated with tuberculosis (p<0.05), including feeling sad recently (29.2% vs. 22.6%), crying more than usual (14.8% vs. 10.7%), frequent headaches (48.5% vs. 43.1%), lack of appetite (49.9% vs. 37.9%), feeling tired easily (65.8% vs. 57.6%), difficulty feeling satisfied with tasks (20.9% vs. 14.0%), feels constantly tired (50.8% vs. 43.9%), feelings of uselessness (7.4% vs. 4.0%) and loss of interest in things (14.4% vs. 9.5%).

**Table 2:** Association between symptoms of common mental disorder and tuberculosis.

Category and Symptoms	Cases (n = 717)		Controls (n = 717)		P-value
	N	Positive %	N	Positive %	
**Depressive/anxious mood**		21.4		23.0	
Feels nervous, tense or worried	324	45.2	342	47.7	0.336
Feels afraid easily	230	32.1	254	35.4	0.168
Has felt sad lately*	209	29.2	162	22.6	0.006
Cries more than usual*	106	14.8	77	10.7	0.035
**Somatic symptoms**		40.9		41.6	
Frequent headaches*	348	48.5	309	43.1	0.041
Poor sleeping	358	49.9	326	45.5	0.168
Uncomfortable feelings in the stomach	230	32.1	250	34.9	0.296
Indigestion	165	23.0	141	19.7	0.104
Lack of appetite*	358	49.9	271	37.9	0.000
Shaking hands	204	28.5	213	29.7	0.684
**Reduction in vital energy**		31.2		30.6	
Feels tired easily*	472	65.8	413	57.6	0.001
Difficulty making decisions	95	13.3	90	12.6	0.987
Difficulty thinking clearly	96	13.4	85	11.9	0.454
Difficulty feeling satisfied with tasks*	150	20.9	100	14.0	0.015
Work/occupation causes suffering	95	13.3	106	14.8	0.301
Feels constantly tired*	364	50.8	315	43.9	0.009
**Depressive thoughts**		6.5		4.8	
Unable to play a useful role in life	61	8.5	51	7.1	0.467
Feels useless in his/her life*	53	7.4	29	4.0	0.019
Has lost interest in things*	103	14.4	68	9.5	0.008
Thinks about ending his/her life	45	6.3	28	3.9	0.099

*p≤0.05.

City of Salvador, Brazil, 2008–2010.

Table 3 shows a statistically significant association, without adjusting for confounders, between overall CMDs and TB (OR: 1.34; 95% CI 1.06–1.68). The magnitude of this association did not change in the multivariate analysis (OR: 1.34; 95% CI 1.05–1.70). The final model was adjusted for diabetes, alcohol abuse, ethnicity, drug use, number of household goods, level of education, history of contact and crowding. Drug use variable was kept in the model because it contributes to the goodness-of-fit, and was considered a confounder a priori because in the literature it is associated with CMD and TB However, this variable did not behave as a confounder of the association under study.

**Table 3:** Association between common mental disorders and tuberculosis.

Variables	Tuberculosis				Crude OR (95% CI)	Adjusted OR^1^ (95% CI)
	Cases		Controls			
**CMD**	N	%	N	%		
Yes	278	38.8	235	32.8	1.34 (1.06–1.68)	1.34 (1.05–1.70)
No	439	61.2	482	67.2	1.0	1.0

1 Adjusted for diabetes, alcohol abuse, ethnicity, drug use, number of household goods, level of education, history of contact and crowding.

City of Salvador, Brazil, 2008–2010.

## Discussion

In this study, a 34% increase in the presence of pulmonary TB was found among individuals with CMDs (OR: 1.34; 95% CI 1.05–1.70). This was the first Brazilian study that investigated the association between CMDs and TB. Our results are consistent with the limited existing literature. A study conducted in Ethiopia with a hospital population found a positive association (OR: 1.7; 95% CI 1.06–1.68) between CMDs and TB in individuals with human immunodeficiency virus (HIV) [9] ^.^ In contrast, our study found an association in a population with an HIV prevalence below 1% [20]. The instruments to diagnose CMDs were different in the two studies: the SQR-20 was used in Salvador, and the Kessler 10 scale was used in Ethiopia [9]. In our study, the use of the SRQ-20 was more appropriate, as the study relied on an outpatient sample.

The link between TB and mental health is potentially complex. Various explanations for the link have been suggested in the literature, including the effects of stress on immune functioning and the progression from infection to disease [12], [13], the occurrence of neuropsychiatric disorders as adverse events of TB treatment [21], and the diagnosis of TB increases risk of mental illness from the stigma and social isolation that are associated with TB [2], [10], [22].

Contrary to other Brazilian studies that found a predominance of “depressive/anxious mood,” the present study found a predominance of “somatic symptoms” among the cases. However, the samples from the previous studies consisted of housewives [23] and homeless [24] individuals. In the present study, symptoms of TB disease may have been captured by the instrument as somatic symptoms of CMDs [5], [21]. “Reduction in vital energy” and “depressive thoughts” may reflect individual stressors; evidence has shown that stress can mediate the relationship between psychosocial problems and physical illness, as stress has an effect on the bacterial load in the lungs and the immune defense system [12], [13].

CMDs receive little attention in medical practice, and tuberculosis is rarely considered in mental health management. The association between mental health and other morbidities contributes to the inadequate adherence to the proposed treatments and aggravates the clinical depiction of both diseases [25], [26]. Additionally, the association between TB and CMDs could reduce adherence or increase the duration of TB treatment, increase the risk of relapse, reduce quality of life and increase public health costs; furthermore, tuberculosis can be misdiagnosed as a CMD in mental health clinics [25], [27]. Poor adherence to antituberculosis medication is an important barrier to global control of the disease, and poor adherence increases the risks of morbidity, mortality, and drug resistance in both individuals and communities [2], [25].

The present study may have limitations. However, classification bias was minimized, as cases and controls were diagnosed by the attending chest physician. Because this study was a case control study and not a cohort study, we cannot establish the direction of the association between CMDs and TB.

## Conclusions

The results of the present study show an association between CMDs and TB. This complex association may result in worse prognoses for TB cases and contribute to the challenges to the global control of TB. Mental health programs should be integrated within TB control programs, and efforts should be made to increase awareness about the high rate of CMDs in TB patients and the implications for TB treatment outcomes. Screening programs for psychological morbidity can be used in primary health units. We recommend that population-based cohort studies of tuberculosis investigate the relationship between CMDs and TB to establish the direction of causality. These studies will further establish the implications for treatment adherence and provide guidance to improve cure rates.
